# Supplementary material for: Causal Relationship between Aging and Anorexia Nervosa: A White-Matter-Microstructure-Mediated Mendelian Randomization Analysis
Source: Biomedicines. 2024 Aug 16;12(8):1874. doi: 10.3390/biomedicines12081874 (PMC11351342; doi:10.3390/biomedicines12081874)
Supplement: Supplementary file 1 [file biomedicines-12-01874-s001.zip › Supplementary Figure/Figure S8/Leaveoneout plot of MR analysis for TL to ubm-b-2039.pdf]

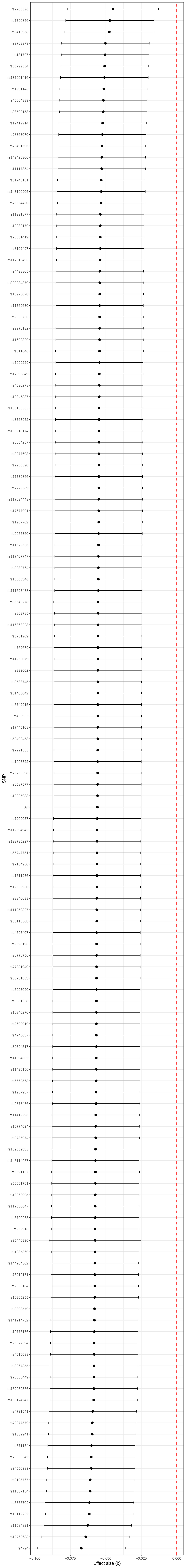
rs7705526
rs7790856
rs9419958
rs2763979
rs131797
rs56799554
rs137901416
rs1291143
rs45604339
rs28502153
rs12412214
rs28363070
rs78491606
rs142426306
rs11117354
rs61748181
rs143190905
rs75664430
rs11991877
rs12932179
rs73581419
rs8102497
rs117512405
rs4498805
rs202034370
rs16978028
rs11769630
rs2056726
rs2276182
rs11699829
rs611646
rs7099229
rs17803849
rs4530278
rs10845387
rs150150565
rs3767952
rs188918174
rs6054257
rs2977608
rs2230590
rs77732866
rs7772289
rs117034449
rs17677991
rs1907702
rs9955360
rs11579626
rs117407747
rs2282764
rs10805346
rs111527438
rs35640778
rs869785
rs116863223
rs6751209
rs762679
rs41269079
rs932002
rs2538745
rs61405042
rs5742915
rs450962
rs17445108
rs59409453
rs7221585
rs1003322
rs73730598
rs6587577
rs12925933
A8
rs7209057
rs112394943
rs139795227
rs55747751
rs7164950
rs1611236
rs12369950
rs9940099
rs111950327
rs80116508
rs4695407
rs9398196
rs6776756
rs77231040
rs66731853
rs6007020
rs6881568
rs10840270
rs9600019
rs4743037
rs80324517
rs41304832
rs11426156
rs6669563
rs1957937
rs9878436
rs11412296
rs10774624
rs3785074
rs139669835
rs145114957
rs3891167
rs56061761
rs13062095
rs117630647
rs6790988
rs939916
rs35446936
rs1985369
rs144204502
rs76219171
rs2555104
rs10905255
rs2293579
rs141214782
rs10773176
rs28577594
rs4616688
rs2967355
rs76666449
rs182059586
rs185174247
rs4731541
rs79977579
rs1332941
rs871134
rs76065543
rs34550383
rs8105767
rs11557154
rs6536702
rs10112752
rs11584821
rs10768683
rs4724
SNP
-0.100
-0.075
-0.050
-0.025
0.000
Effect size (b)
